# Nigratine as dual inhibitor of necroptosis and ferroptosis regulated cell death

**DOI:** 10.1038/s41598-022-09019-w

**Published:** 2022-03-24

**Authors:** Claire Delehouzé, Arnaud Comte, Stephen Adonai Leon-Icaza, Céline Cougoule, Marcelle Hauteville, Peter Goekjian, Jeannette Chloë Bulinski, Marie-Thérèse Dimanche-Boitrel, Etienne Meunier, Morgane Rousselot, Stéphane Bach

**Affiliations:** 1 SeaBeLife Biotech, Place Georges Teissier, 29680 Roscoff, France; 2 Sorbonne Université, CNRS, UMR 8227, Integrative Biology of Marine Models Laboratory (LBI2M), Station Biologique de Roscoff, 29680 Roscoff, France; 3 Université de Lyon, CNRS UMR 5246, ICBMS, Chimiothèque, Université Claude Bernard Lyon 1, 69622 Villeurbanne, France; 4 Institut de Pharmacologie et Biologie Structurale (IPBS), Université de Toulouse, CNRS, UPS, Toulouse, France; 5 Laboratoire de Biochimie Analytique et Synthèse Bioorganique, Université de Lyon, Université Claude Bernard Lyon 1, 69622 Villeurbanne, France; 6 Université de Lyon, CNRS UMR 5246, ICBMS, Laboratoire Chimie Organique 2-Glycosciences, Université Claude Bernard Lyon 1, 69622 Villeurbanne, France; 7 Department of Biological Sciences, Columbia University, New York, NY 10027 USA; 8 Univ Rennes, Inserm, EHESP, Irset (Institut de Recherche en Santé, Environnement et Travail) - UMR_S 1085, 35000 Rennes, France; 9 Sorbonne Université, CNRS, FR 2424, Plateforme de Criblage KISSf (Kinase Inhibitor Specialized Screening Facility), Station Biologique de Roscoff, 29680 Roscoff Cedex, France; 10 Centre of Excellence for Pharmaceutical Sciences, North-West University, Private Bag X6001, Potchefstroom, 2520 South Africa

**Keywords:** Chemical tools, Drug development

## Abstract

Nigratine (also known as 6E11), a flavanone derivative of a plant natural product, was characterized as highly specific non-ATP competitive inhibitor of RIPK1 kinase, one of the key components of necroptotic cell death signaling. We show here that nigratine inhibited both necroptosis (induced by Tumor Necrosis Factor-α) and ferroptosis (induced by the small molecules glutamate, erastin, RSL3 or cumene hydroperoxide) with EC_50_ in the µM range. Taken together, our data showed that nigratine is a dual inhibitor of necroptosis and ferroptosis cell death pathways. These findings open potential new therapeutic avenues for treating complex necrosis-related diseases.

## Introduction

Advances in systems biology have revealed that single-target compounds are less efficient in preventing or curing complex diseases such as neurodegenerative diseases^1,2^. Drug discovery failures in complex cases, including Alzheimer's disease (AD), suggest that the definition of a “magic bullet” (i.e., drug selective for a single molecular target), the scientific concept developed by Paul Ehrlich more than a century ago^3^, may need to be revised. The development of multi-target-directed ligands (MTDLs) for AD treatment is a notable example of promising therapeutic strategies^2^. Moreover, the central nervous system (CNS) drugs most effective clinically, such as clozapine for treatment of schizophrenia, act as “magic shotguns”: non-selective drugs with pleiotypic actions^4^. These compounds are effective in treating complex human disorders because they are able to modulate multiple targets involved in the pathophysiological processes, a strategy called polypharmacology. Synergistic effects are also among the main advantages of this combined therapeutic approach^5^. This strategy is now widely used in drug development as evidenced by the fact that, from 2015 to 2017, 21% of the new molecular entities (NMEs) approved by the food and drug administration (FDA) are multi-target drugs (by contrast, 34% were single-target small molecules)^6^.

The systemic breakdown of physiological networks in disease is not restricted to brain tissue; it is observed in numerous other tissues, including kidneys and liver. Functional redundancy has been described for numerous cellular pathways and is well-described for regulated cell death (RCD) signaling^7^. Indeed, in RCD signaling, redundancy may reflect the evolutionary history of cell suicide pathways, wherein autophagic, apoptotic or necrotic elements might have arisen from an ancestral cell death mechanism. Ancestral cell death routes probably include ferroptosis, a non-apoptotic cell death that is catalyzed by iron^8^, as ferroptosis-like pathway exists in cyanobacteria, phytoplankton that evolved over 2.7 billion years ago^9^. There are at least 12 distinct types of RCD including various forms of regulated necrosis^7^. Since the term “regulated” indicates a reliance on fine-tuned molecular signaling machinery, it is possible to screen for small chemical compounds that can modulate the signaling cascades and effectors in RCD pathways (such as necrostatins that block necroptosis^10^).

Small molecule inhibitors of RCD pathways are potential new therapeutic approaches to pathologies such as inflammatory and neurodegenerative diseases^11^. Necroptosis is a regulated cell necrosis route that can be activated under apoptosis-deficient conditions. Necroptosis depends notably on the serine/threonine kinase activity of RIPK1 (Receptor-Interacting Protein Kinase 1) and RIPK3 and on the trafficking and accumulation at the plasma membrane of the pseudokinase MLKL (Mixed lineage kinase domain-like)^11,12^. As of March, 2021, four RIPK1 inhibitors had reached clinical trials (GSK2982772 and GSK3145095 developed by GSK, DNL747 (SAR 443060) and DNL758 (SAR443122) developed by Sanofi and Denali Therapeutics, respectively), and GFH312 developed by GenFleet Therapeutics, for treatments of psoriasis (phase II), ulcerative colitis (phase II), rheumatoid arthritis (phase II), or pancreatic cancer (phase II), for the molecules developed by GSK; Alzheimer’s disease (phase I), cutaneous lupus erythematosus (phase II, *not yet recruiting*), amyotrophic lateral sclerosis and multiple sclerosis (phase I), and severe coronavirus caused by SARS-Co-V-2 (COVID-19) for the molecules developed by Sanofi and Denali Therapeutics; and for evaluation of the safety/tolerability and pharmacokinetics in healthy subjects (phase I, *not yet recruiting*) for the molecule developed by GenFleet Therapeutics (data from clinicaltrials.gov and^13^). Numerous other compounds (40 +) also have demonstrated binding affinity for RIPK1^13^. Recently, Tonnus et al*.* generated a combined small molecule inhibitor (Nec-1f) that simultaneously targets RIPK1 and ferroptosis. Nec-1f was shown to improve survival in models of ischemia–reperfusion injury^14^.

Our research group has contributed to the characterization of synthetic and natural product derivatives that are potent inhibitors of RIPK1, including the 7-azaindole derivative Sibiriline^15^, the marine-derivative 2-aminobenzothiazole MBM105^16^ and the flavanone nigratine (also known as 6E11)^17^. Nigratine (2-(4-(benzyloxy)phenyl)-2,5-dihydroxy-7-methoxychroman-4-one) is a synthetic derivative of the naturally occurring 2,5-dihydroxy-2-phenylchroman-4-ones isolated from *Populus nigra* buds. We previously showed that nigratine is a non-ATP competitive inhibitor with a remarkable selectivity toward RIPK1 and is able to protect human aortic endothelial cells (HAEC) from cold hypoxia/reoxygenation-induced cell death^17^.

We now report the characterization of nigratine as new inhibitor of the ferroptosis RCD pathway (with EC_50_ in the µM range). Nigratine is thus considered as a new dual inhibitor (or “magic shotgun”) of both necroptosis and ferroptosis RCD death that can be used in polypharmacological approaches for treatments of regulated-necrosis related disorders.

## Results

### Effect of nigratine on necroptotic cell-death induced by TNF-α

**Figure 1:**
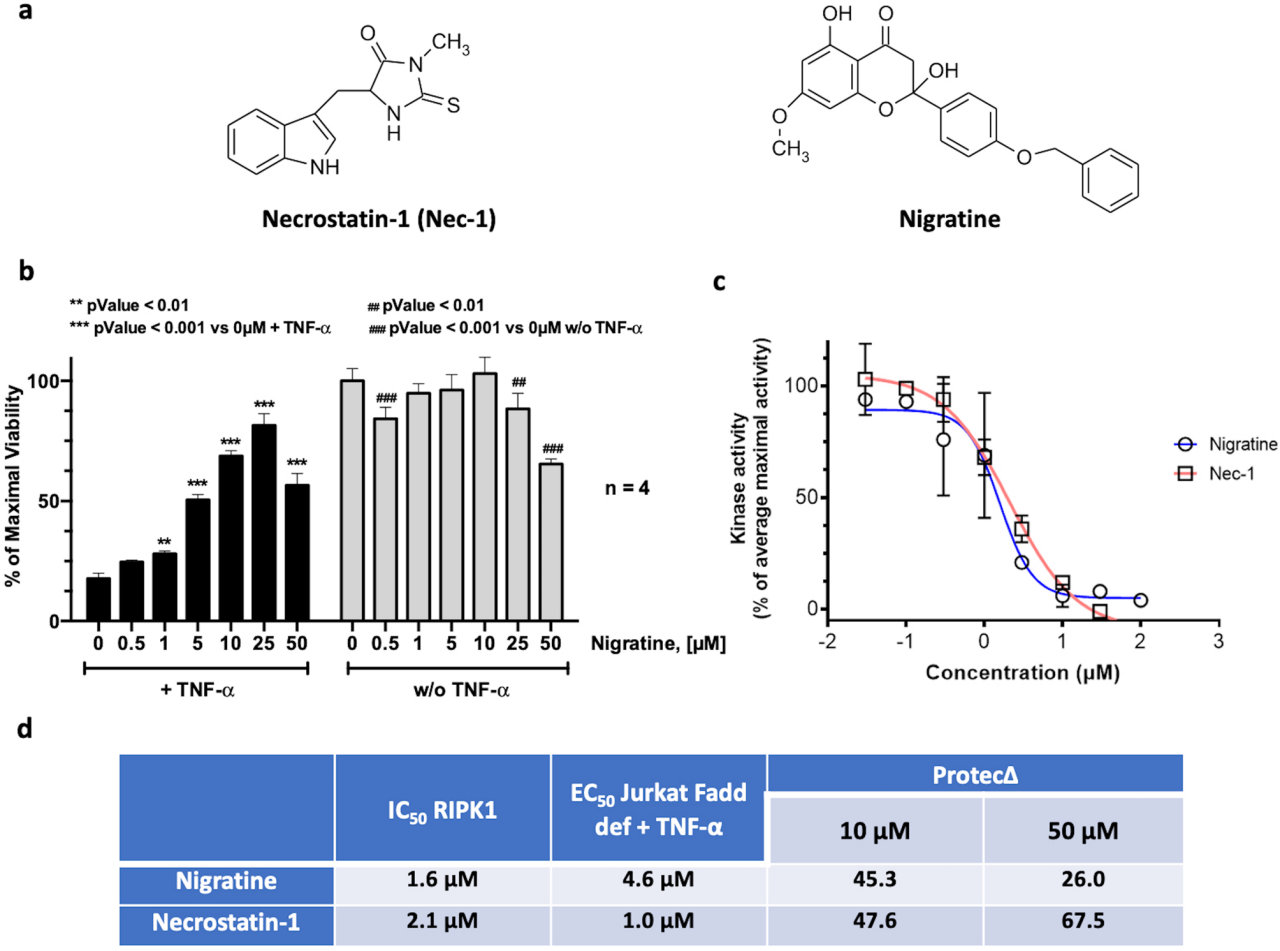
Nigratine inhibits necroptosis by targeting RIPK1 kinase. (**a**) Nigratine and necrostatin-1 (Nec-1), as control inhibitor of necroptosis, are structurally non related molecules. (**b**) Jurkat-Fadd def cells were treated 24 h with increasing concentrations of nigratine alone or co-treated with 10 ng/ml of TNF-α. Cell viability was estimated by MTS assay. Data are shown as the mean ± SD of four replicates. (**c**) The RIPK1 catalytic activity was monitored using myelin basic protein, MBP, as a substrate. RIPK1 was assayed in the presence of increasing concentrations of both nigratine and Nec-1. The kinase activities are expressed in % of average maximal activity, i.e. measured in the presence of DMSO (n = 2, mean ± SEM). (**d**) The IC_50_ (on kinase) and EC_50_ and “protection delta” (ProtecΔ) (on cells) values were determined from the dose–response curves using Graphpad PRISM software and reported in the table. The level of protection against the TNF-α-induced cell death is estimated using the ProtecΔ values (first described and used in^16^). These data are determined by subtracting the value obtained for cells only treated with TNF-α from the value obtained for co-treatment with TNF-α and with the tested compound.

Nigratine was characterized by Delehouzé et al. as new necroptosis inhibitor using a TNF-induced FADD-deficient human Jurkat necroptosis assay^17^. TNF-α can induce necroptosis in Jurkat cells when FADD is deleted. We used an MTS assay to measure the viability of lymphocytes and we determined nigratine effects on RIPK1-kinase activity, in order to elucidate the mechanism of necroptosis inhibition. In this study, we compared the bioactivity of nigratine to that of necrostatin-1 (Nec-1), a known inhibitor of necroptosis^10^. Both compounds were tested at concentrations of 10 and 50 µM. The results obtained and the chemical structures of these compounds are depicted in Fig. 1. In this assay, nigratine inhibited both necroptosis cell death and RIPK1 kinase activity with an activity similar to that of Nec-1 (with both EC_50_ and IC_50_ in the μM range). The value of the protection index (dubbed ProtecΔ) for nigratine at 10 µM (45.0) compared to the value at 50 µM (26.0) suggested that in Jurkat FADD-deficient cells the higher concentration produced a lesser effect.

### Nigratine protects neuronal cells against ferroptotic cell death

In Delehouzé et al., we previously showed that treatment of human aortic endothelial cells (HAEC) with nigratine during cold hypoxia or during reoxygenation following cold hypoxia conferred measurable benefits on cell survival. Nigratine was significantly more potent than the specific RIPK1 necroptosis inhibitor, Nec-1s^17^. This result suggested that the observed effect of nigratine was not fully attributable to its inhibition of RIPK1 kinase. Indeed, when compared to nigratine, Nec-1s is a more potent inhibitor of RIPK1^17^.

**Figure 2:**
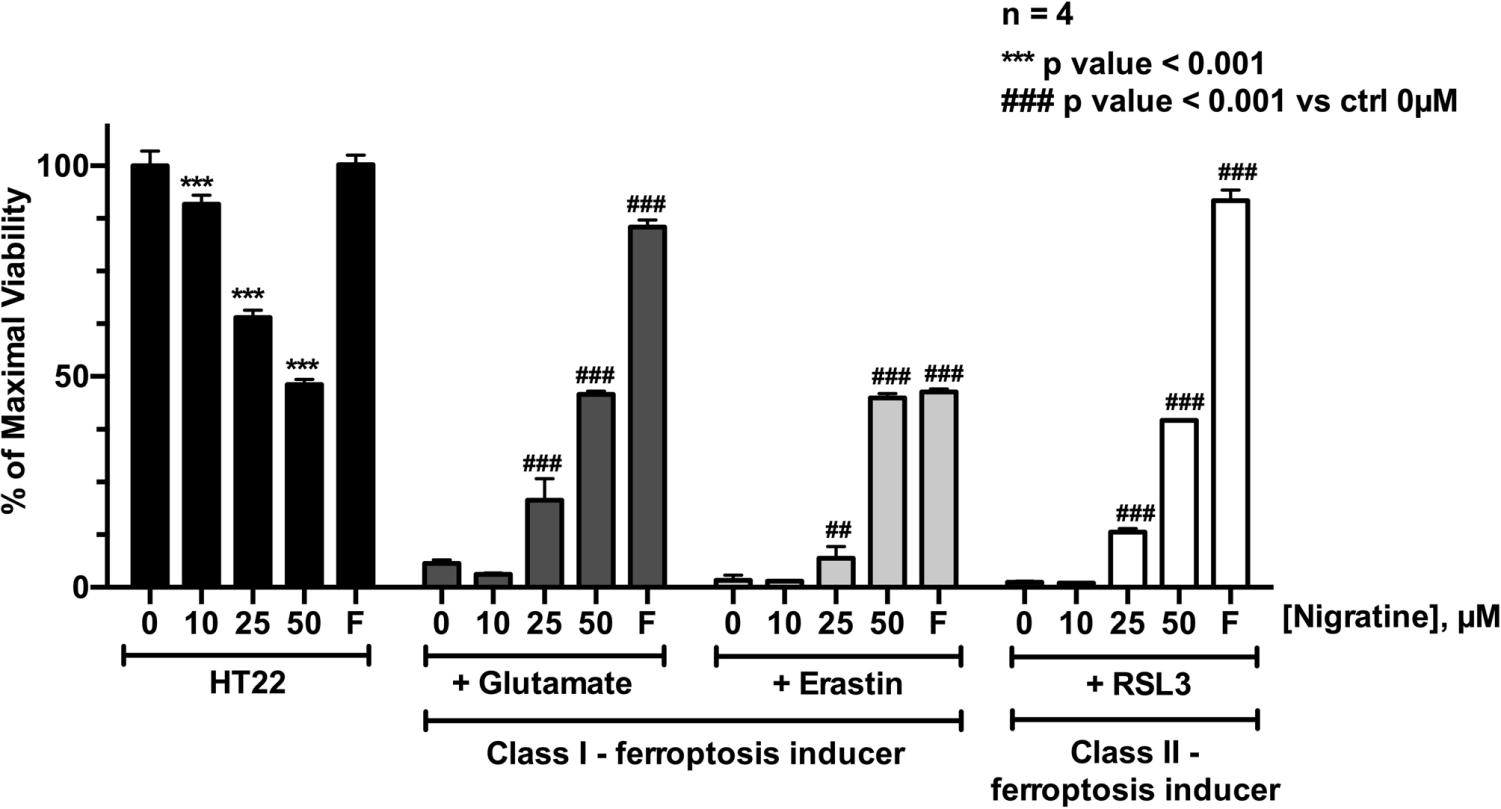
Nigratine protects HT-22 mouse hippocampal neuronal cell line from cell death triggered by both class I and II-inducers of ferroptosis. HT22 cells were treated 24 h with increasing concentrations (0–50 µM) of the tested chemical compounds alone (“HT22”) or co-treated with 5 mM glutamate, 0.5 µM erastin or 1 µM of RSL3. Ferrostatin-1 (“F”) was used as a positive control for ferroptosis inhibition. Cell viability was estimated by MTS assay. Data are shown as the mean ± SD of four replicates.

**Figure 3:**
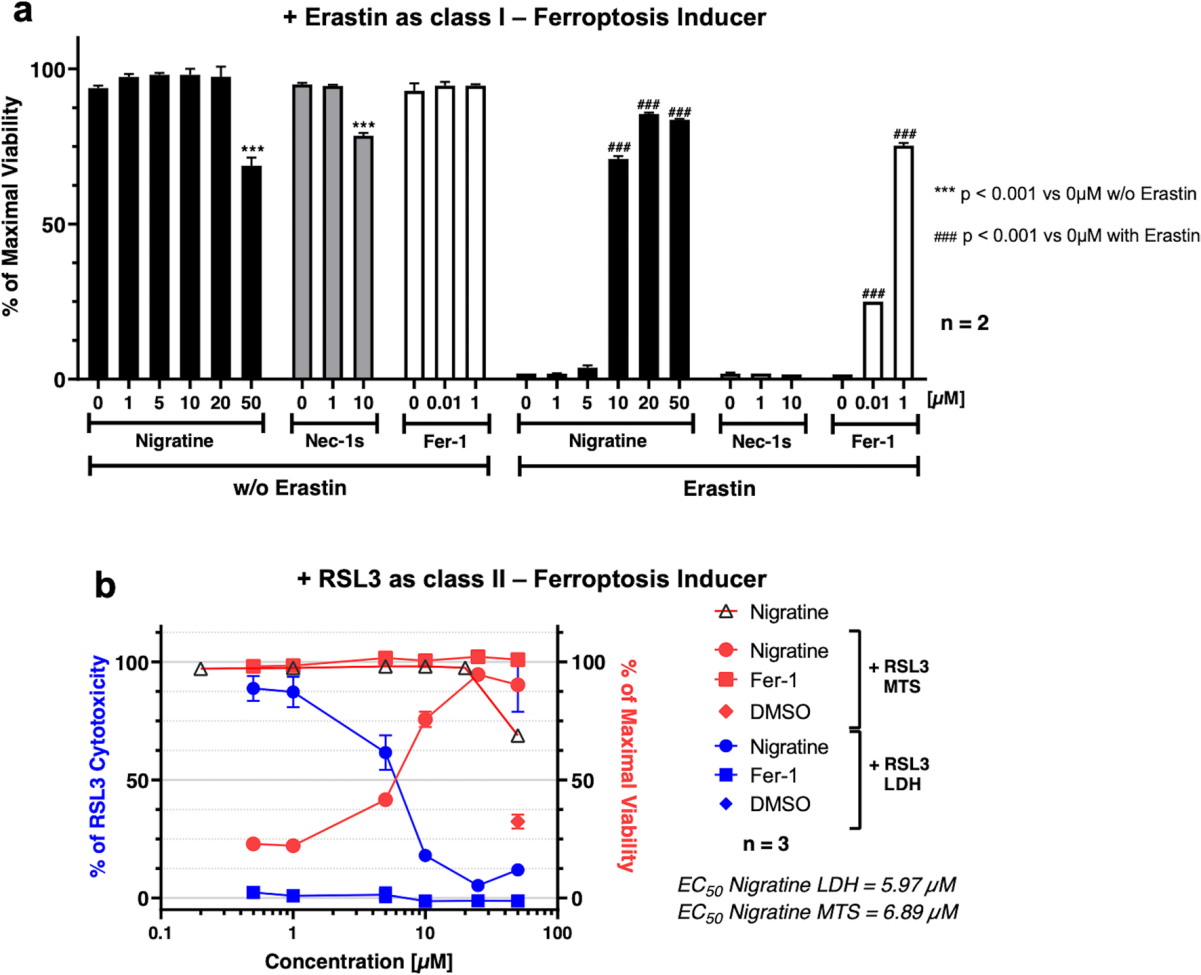
Nigratine protects SH-SY5Y human neuroblastoma cell line from cell death triggered by both class I and II-inducers of ferroptosis. (**a**) SH-SY5Y cells were co-treated 24 h with various concentrations of the tested chemical compounds alone and 10 µM of erastin. Ferrostatin-1 (Fer-1) was used as a positive control for ferroptosis inhibition. Cell viability was estimated by MTS assay. Data are shown as the mean ± SEM of two replicates. (**b**) SH-SY5Y cells were treated 24 h with 5 µM of RSL3 and increasing concentrations of nigratine or ferrostatin-1 (Fer-1). Cell death was evaluated by lactate dehydrogenase (LDH) release assay. Results are plotted in % of LDH release measured when cells are treated with RSL3 (left axis, colored blue). Cell viability was measured by MTS reduction assay. The results obtained (colored red) were plotted in % of maximal viability (detected in DMSO-treated cells, right axis). Data are shown as the mean ± SD of three replicates.

We next analyzed the effect of nigratine treatment on ferroptotic cell-based models. Ferroptosis was first characterized in *NRAS*-mutant HT-1080 fibrosarcoma cells treated with erastin^8^. Erastin, like excess L-glutamate (5 mM), is a class I ferroptosis inducer. Both compounds inhibit cystine uptake by the heterodimeric cystine/glutamate antiporter (system xc −), thus inducing oxidative toxicity. This antiporter is a key component essential for protection of cells from oxidative stress and lethal lipid peroxidation, both of which are major hallmarks of ferroptosis. Glutathione peroxidase (GPX4) is also a key player of ferroptosis. The execution of ferroptosis depends on massive lipid peroxidation, which can be reduced by the lipid repair enzyme, GPX4. RSL3 belongs to class II of the ferroptosis-inducing agents by virtue of its inhibition of GPX4^18^. Accordingly, we tested the protective effect of nigratine against class I (glutamate and erastin) and II (RSL3) ferroptosis inducers in two neuronal cell lines, mouse HT22 (hippocampal neuronal cell line) and in human SH-SY5Y (neuroblastoma cell line). These cell lines have been widely used as models of neurodegeneration, neurotoxicity, and neuroprotection^19,20^. As shown on Figs. 2 and 3, 50 µM nigratine was able to strongly inhibit the ferroptotic cell death induced by excess of glutamate, erastin, and RSL3, in both murine HT22 and human SH-SY5Y cell lines.

The dose-dependent effect of nigratine on cell viability was analyzed using an MTS assay. In order to validate the effect of nigratine on neuroprotection and necrosis, the extracellular lactate dehydrogenase (LDH) detection assay was also used as an independent cell death assay^21^. This assay measures LDH that is released into the extracellular space when the plasma membrane is damaged; this release is characteristic of necrotic cell death^22^. As shown in Fig. 3b, the treatment of SH-SY5Y cells with nigratine protected cells against cell-death in a dose-dependent manner, correlated with the dose-dependent increase in cell survival. Our data also showed that nigratine had a negligible cytotoxic effect on SH-SY5Y neuroblastoma cells when applied at ≤ 10 µM (Fig. 3b). Interestingly, nigratine is active against ferroptosis on SH-SY5Y cells with an EC_50_ of ~ 6.5 µM (EC_50(LDH)_ = 6.0 µM and EC_50(MTS)_ = 6.9 µM) similar to its activity in preventing necroptosis (EC_50(MTS)_ = 4.6 µM).

### Nigratine inhibits phospholipid peroxidation

**Figure 4:**
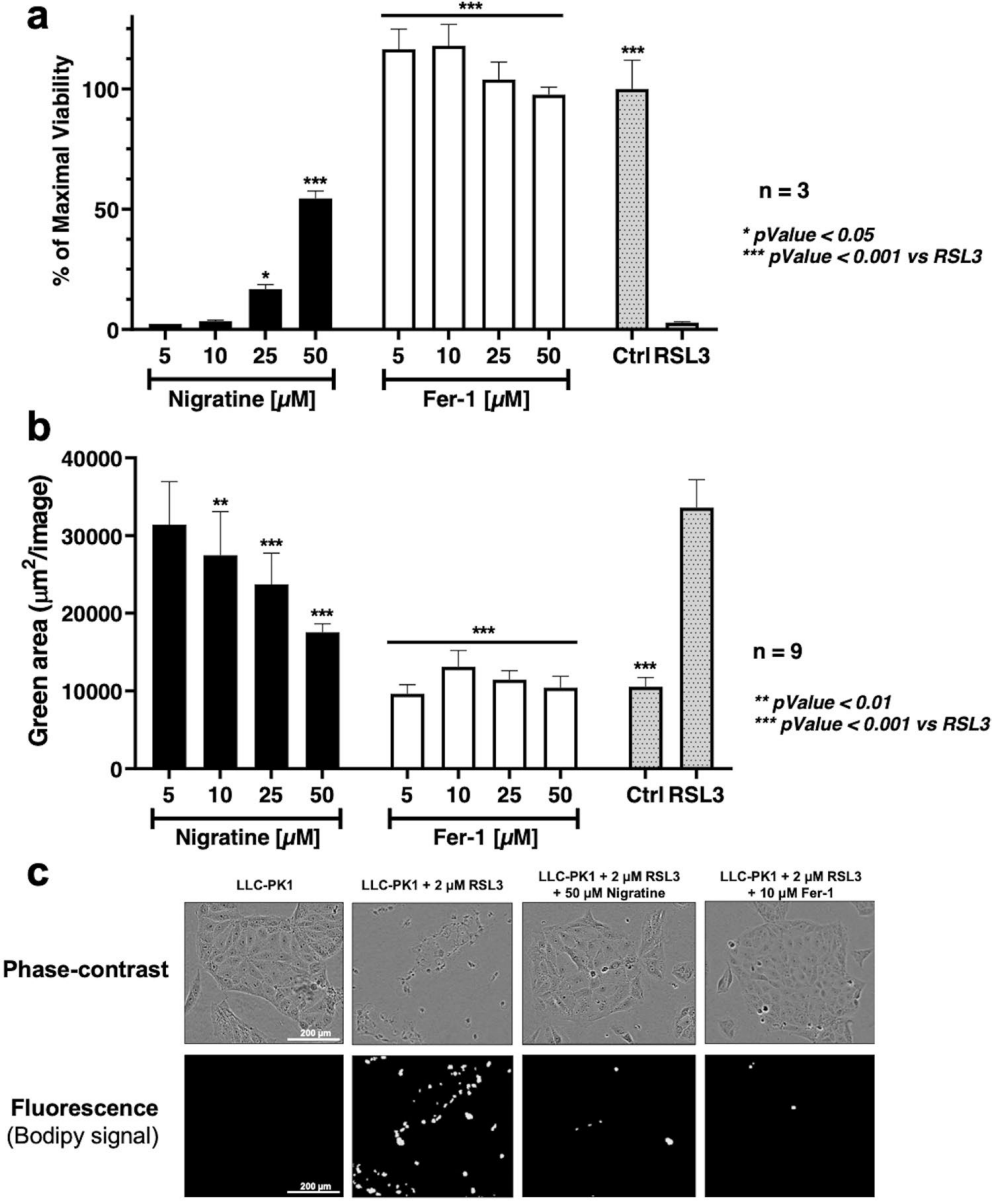
Nigratine protects porcine kidney epithelial LLC-PK1 cell line from lipid peroxidation and cell death triggered by RSL3. LLC-PK1 cell line was treated with 2 µM of RSL3 and increasing concentration of nigratine or ferrostatin-1 (Fer-1). (**a**) Cell viability was estimated by MTS assay. Data are shown as the mean ± SD of three replicates. (**b**) Lipid peroxidation was detected by cellular BODIPY 581/591 C11 staining. Fluorescence was recorded with the IncuCyte S3 live cell imaging apparatus. Data are shown as the mean ± SD of three replicates of nine replicates. (**c**) Representative phase-contrast and fluorescence images of cells stained with BODIPY 581/591 C11 probe are visualized using the IncuCyte S3 live cell imaging apparatus.

We next characterized the effect of nigratine on the peroxidation of phospholipids induced by the inhibition of GPX4 with RSL3; for this study we selected the well-characterized porcine renal epithelial proximal tubule cell line (LLC-PK1). As shown in Fig. 4, our results showed that: (i) nigratine significantly protects renal epithelial cells against ferroptotic cell death induced by RSL3 (Fig. 4a) and (ii) nigratine inhibits phospholipid peroxide formation detected in cells using BODIPY 581/591 C11 probe (Fig. 4b,c). The BODIPY dye was used as a sensitive indicator of free radical processes that have the potential to oxidize lipids within membranes. Taken together, these results suggested that nigratine has a marked effect on lipid peroxidation, a major hallmark of ferroptosis.

### Nigratine is a weak antioxidant compound

**Figure 5:**
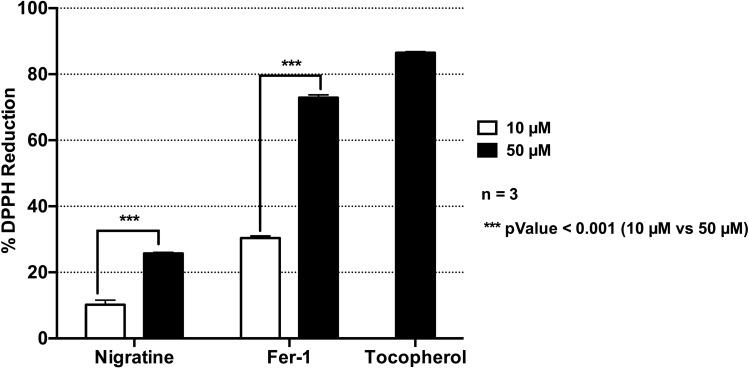
Antioxidant activity of nigratine. DPPH reduction assay was conducted for 30 min at room temperature after the addition of a DPPH solution at 15.85 µM (in 90/10, vol/vol, methanol/water) in 96-well plate. Reduction of DPPH was determined by absorbance measurement at 517 nm. Tocopherol (vitamin E), a well-known antioxidant, and Fer-1, as lipophylic antioxidant, were used as a positive controls. Data are shown as the mean ± SD of three replicates.

Ferroptosis, catalyzed by iron, comprises a failure of the glutathione-dependent antioxidant defenses that result from a loss of activity of the lipid repair enzyme, glutathione peroxidase 4 (GPX4)^23^. This in turn leads to an accumulation of lipid-based reactive oxygen species (ROS), and lipid peroxidation. Antioxidant compounds such as α-tocopherol (vitamin E), a potent radical-trapping antioxidant that blocks the auto-oxidation of chain-propagating peroxyl radicals and protects hydrocarbon biological systems from oxidation and membrane damage in ferroptosis, have been shown to inhibit ferroptotic RCD (see^24^ for review). Our results (Fig. 5) suggest that nigratine is only a weak antioxidant compound compared to α-tocopherol and the lipophilic antioxidant ferrostatin-1.

### Nigratine protects human bronchial organoids against both ferroptotic and necroptotic cell death

**Figure 6:**
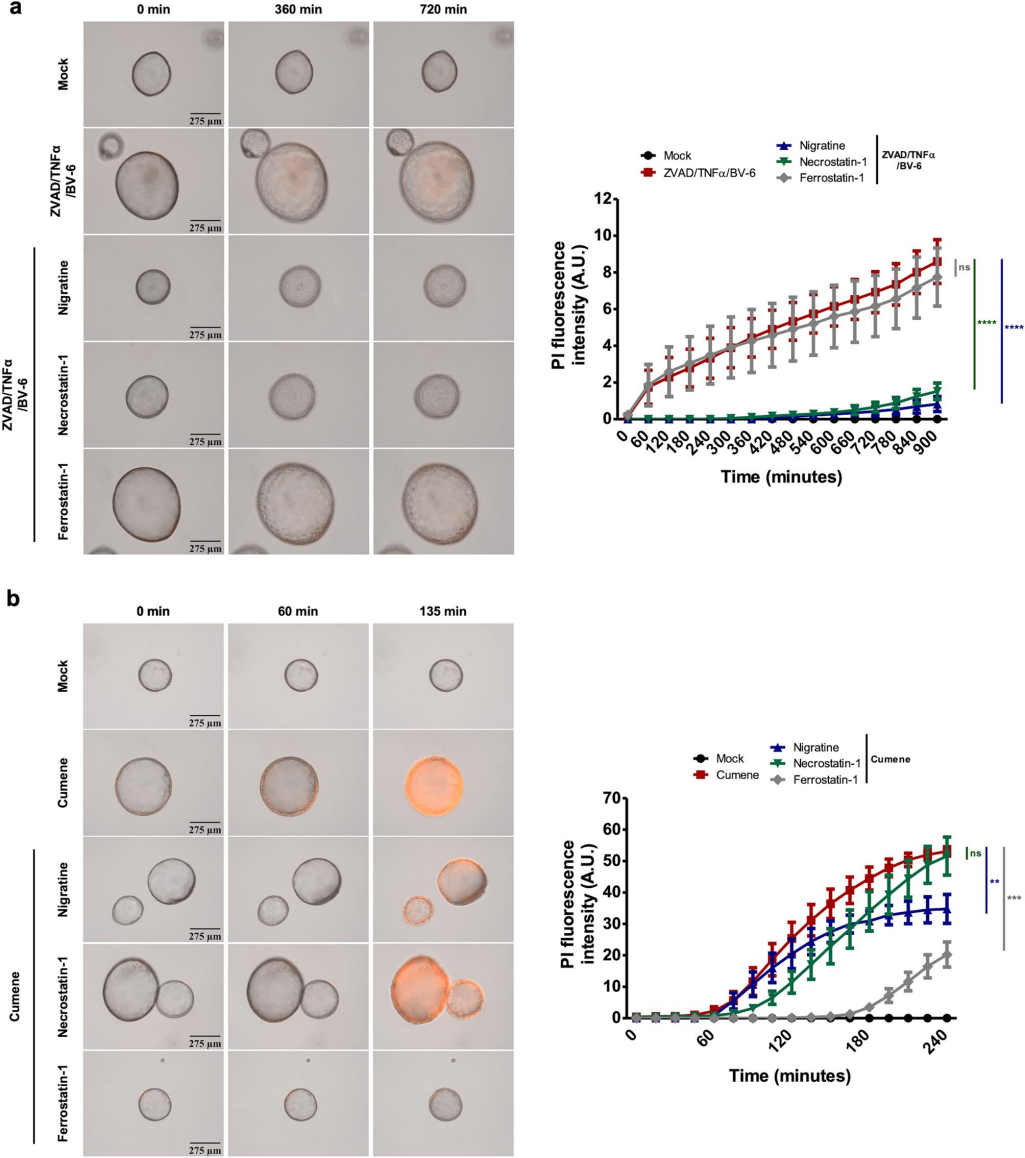
Nigratine protects human bronchial organoids from necroptosis and ferroptosis. (**a**) Time-lapse microscopy and the associated quantifications of the measure of plasma membrane permeabilization using propidium iodide incorporation in human primary bronchial organoids stimulated with the mix of 40 μM Z-VAD, 100 ng/ml Human TNF-α and 5 µM BV-6 in presence or absence of 25 μM nigratine, 40 μM necrostatin-1 or 40 μM ferrostatin-1 for 15 h. Data are plotted as means + /− SEM. pValue calculated by *t*-test. (**b**) Time-lapse microscopy and the associated quantifications of the measure of plasma membrane permeabilization using propidium iodide incorporation in human primary bronchial organoids stimulated with 800 µM of cumene hydroperoxide in presence or absence of 25 μM nigratine, 40 μM necrostatin-1 or 40 μM ferrostatin-1 for 4 h. Data are plotted as means +/− SEM. pValues were calculated by *t*-test (**pValue < 0.01, ***pValue < 0.001, ****pValue < 0.0001).

To determine in a more complex system the dual ability of nigratine to inhibit both necroptosis and ferroptosis, we exploited human bronchial organoids from healthy patients and exposed them to activators of necroptosis (Z-VAD/TNF-α/BV-6) or ferroptosis (Cumene Hydroperoxide, CuOOH). We monitored propidium iodide (PI) incorporation as a marker of plasma membrane destabilization over time. Our results showed a strong inhibitory effect of nigratine and necrostatin-1 against Z-VAD/TNF-α/BV-6-induced PI incorporation in lung organoids (Fig. 6). The study of CuOOH-induced ferroptosis of bronchial human organoids showed that ferrostatin-1 and nigratine inhibited and delayed PI incorporation, suggesting that nigratine exhibits some anti-ferroptotic properties. Taken together, our results suggest that nigratine has a dual effect against ferroptosis and necroptosis in primary human organoids.

## Discussion

Nigratine was previously described as a putative non-ATP competitive type III RIPK1 kinase inhibitor that can block the necroptosis RCD pathway in various cellular models, including Jurkat lymphocytes, with an EC_50_ in the µM range^17^. Necroptosis is clearly distinct from apoptosis as it does not involve the key apoptosis regulators, such as caspases or cytochrome c release from mitochondria. In the present study, we showed that nigratine also inhibits ferroptosis. For example, nigratine suppressed cell death induced by class I, II and IV ferroptosis inducers; namely, the presence of excess glutamate, erastin, RSL3 and cumene hydroperoxide, respectively. We also showed that nigratine inhibits phospholipid peroxidation induced by RSL3 in epithelial LLC-PK1 porcine renal cells, an inhibition that was also observed for the known ferroptosis inhibitor, ferrostatin-1. The dual protective effect of nigratine against both necroptosis and ferroptosis was also observed in human bronchial organoids.

Polypharmacological approaches, that is, use of drugs that act on multiple targets, appears well-suited to improving the outcome of complex diseases. Thus, nigratine would seem to be very attractive as a therapy for preventing and/or treating disorders associated with the induction of necroptosis and/or ferroptosis. As an example, the therapeutic benefit of nigratine could be evaluated in animal models of cisplatin-induced nephrotoxic acute kidney injury (AKI), in which both regulated RCD pathways are known to be involved in proximal tubular cell death^25–27^. Ischemia/reperfusion injuries (IRI), which have been attributed to cell necrosis for decades^28^, should also be explored with nigratine.

Canonical ferroptosis inhibitors like the lipophilic antioxidant, ferrostatin-1, prevent the accumulation of toxic lipid and cytosolic ROS, inhibiting ferroptotic cell death. As observed here using the DPPH assay, nigratine is comparatively less potent as an antioxidant than the two aromatic amines, ferrostatin-1 and α-tocopherol. We consequently hypothesize that nigratine does not use a mechanism linked to peroxyl radical scavenging for inhibiting ferroptosis. Alternatively, nigratine may inhibit another cellular target that concomitantly contributes to the overall protection from RCD. Future work will be carried out to identify the molecular target(s) of nigratine in ferroptosis, using various approaches such as target fishing (see^29^ for review). Identification of nigratine’s molecular target(s) is a prerequisite for improving the efficacy of multi-target inhibitors on ferroptosis-related phenotypes, without affecting the activity of its primary target, the receptor-interacting protein kinase 1 (RIPK1). Numerous approaches are available to increase the efficacy of this class of drugs via rational drug design. These approaches include the combination of medicinal chemistry and computational strategies that has been successfully applied to dual-target kinase inhibitors^30^.

Taken together, our results on nigratine indicate that pharmaceutical agents acting on both necroptosis and ferroptosis RCD pathways can be engineered for use in treating complex diseases that involve the activation of multiple RCD pathways. Our study sheds light on the emergence of polypharmacological approaches for treating multiple disorders in which necrosis is of central pathophysiological relevance, such as: ischemia–reperfusion injury in brain, heart and kidney, inflammatory diseases, sepsis, retinal disorders and neurodegenerative diseases.

## Methods

### Cell lines and culture

SH-SY5Y and HT22 cell lines were obtained from American Type Culture Collection (ATCC, Manassas, VA, USA) and were maintained in Dulbecco’s modified eagle’s medium (DMEM) containing 10% fetal bovine serum (FBS) in a humidified atmosphere of 5% CO_2_ at 37 °C. LLC-PK1 was obtained from the European Collection of Authenticated Cell Cultures (ECACC, Porton Down, Salisbury, UK) and maintained in M199 medium supplemented with 10% FBS and cultured in humidified atmosphere at 37 °C under 5% CO_2_. Medium and serum were purchased from Thermo Fisher Scientific (Gibco, Waltham, MA, USA).

### Reagents

Necrostatin-1 (Nec-1) and necrostatin-1s (Nec-1s) were from Calbiochem (VWR International, Fontenay-sous-Bois, France), Z-VAD was from Invivogen (Toulouse, France), Ferrostatin-1 (Fer-1), BV-6, Ras-selective lethal small molecule (RSL3) were from Selleckchem (Houston, TX, USA), 2,2-Diphenyl-1-picrylhydrazyl (DPPH), cumene hydroperoxide and α-tocopherol (vitamin E) were from Sigma Aldrich (St. Louis, MO, USA). Human TNF-α was obtained from Invitrogen (Carlsbad, CA, USA). This cytokine was used in the cell-based assay for the characterization of necroptosis inhibitors. Nigratine was obtained from SeaBeLife Biotech (Roscoff, France). The chemical synthesis of nigratine was described by Hauteville et al^31^.

### Cell death and cell viability assays

SH-SY5Y and HT22 cells were seeded in 96-well plates at a density of 10 000 or 5000 cells per well respectively, following overnight incubation. Cells were treated with 5 µM (SH-SY5Y cells) or 1 µM (HT22 cells) of RSL3; 10 µM (SH-SY5Ycells) or 0.5 µM (HT22 cells) of erastin or 5 mM (HT22 cells) of glutamate and increasing concentrations of compounds for 24 h (100 µl per well).

Cell death was determined by measurement of lactate dehydrogenase (LDH) leakage using the LDH Cytotoxicity assay kit (Invitrogen, Carlsbad, CA, USA) following manufacturer’s recommendations. LDH is a cytosolic enzyme that is rapidly released into the supernatant after cell damage. After 24 h of treatment 50 µl of supernatants were transferred into a clean 96-well plates, reagents were added and LDH activity was measured using a microplate reader. Calculation of cytotoxicity was determined by dividing the LDH activity of each compounds with RSL3 by that of DMSO with RSL3, then multiplying the result by 100.

Cell viability was assessed by MTS assay (CellTiter 96® AQueous Non-Radioactive Cell Proliferation Assay; Promega, Fitchburg, WI, USA) according to the manufacturer’s instructions. This assay is based in the reduction of the 3-(4,5-dimethylthiazol-2-yl)-5-(3-carboxymethoxyphenyl)-2-(4-sulfophenyl)-2H-tetrazolium (MTS) by viable cells to form a colored formazan product. After treatment, cells were incubated 3 h at 37 °C, 5% CO_2_ with MTS. The absorbance was measured using a microplate reader at 490 and 630 nm and the percentage of viability was calculated by dividing the absorbance of testing compound by the absorbance of DMSO treated cells (control).

### Human bronchial organoid production and maintenance

Airway organoids were derived from healthy lung tissue of donors receiving surgical treatment for non-small cell lung carcinoma (NSCLC) as described^32,33^. Human lung tissue was provided by the university hospital (CHU of Toulouse, France), according to the CNRS-approved protocol, CHU 19 244 C and Ref CNRS 205,782. An "informed consent to scientific use of their material" was obtained from all patients (or, if participants are under 18, from a parent and/or legal guardian) participating in this study. All methods were performed in accordance with the relevant guidelines and regulations. The organoids were passaged every 4 weeks.

### Airway organoids stimulation

Before stimulation, 40 µl drops of Cultrex growth factor reduced BME type 2 (R & D Systems) containing airway organoids were seeded on Nunclon Delta surface 24‐well plates (Thermo Scientific) and 500 µl of Advanced DMEM/F12 (Invitrogen) supplemented with 1 × L-Glutamine (Fisher Scientific) and 10 mM Hepes (Fisher Scientific) was added to each plate. Depending on the indicated conditions, organoids were pretreated or no with 25 μM nigratine (SeaBeLife Biotech, Roscoff, France), 40 μM necrostatin-1 or 40 μM Ferrostatin-1 for 1 h before stimulation. All the inhibitors were maintained throughout the experiment. After pretreatment, the organoids were stimulated to induce necroptosis with the mix of 40 μM Z-VAD, 100 ng/ml Human TNF-α and 5 µM BV-6 or to trigger ferroptosis with 800 µM of cumene hydroperoxide. For time-lapse imaging, stimulated organoids were stained with 50 μg/mL Propidium Iodide (Thermo Scientific). Images were acquired every 15 min for the duration of experiments under an EVOS M7000 (Thermo Scientific) Imaging System (10 × objective, organoids were incubated at 37 °C with 5% CO_2_). Data was analyzed using the open source image processing program Fiji/ImageJ (https://imagej.nih.gov/ij/index.html).

### RIPK1 kinase assay

Human RIPK1 full length GST-tagged was purchased from SignalChem (Richmond, CA, USA). The protocol used to detect the enzymatic activity was described in Delehouzé et al.^17^. Briefly, RIPK1 kinase was performed on KISSf Screening Facility (IBISA, Biogenouest, Station Biologique, Roscoff, France) with myelin basic protein (MBP, Sigma, #M1891) as substrate and in the presence of 15 µM ATP.

### Cell-based lipid peroxidation assay

Ferroptosis was induced by treatment of porcine LLC-PK1 cells (derived from the renal epithelial cells of Hampshire pigs PK1) with 2 µM of RSL3. For lipid peroxidation assay, cells were seeded in 96-well black plates with clear bottom at a density of 10,000 cells per well. The assay was performed as previously described by Kahn-Kirby et al.^34^. Briefly, after overnight incubation, cells were pre-labeled with 10 µM of BODIPY 581/591 C11 dye (Invitrogen, Carlsbad, CA, USA) for 30 min at 37 °C; 5% CO_2_. Cells were washed 3 times with PBS before adding treatment. Cells were then treated with RSL3 (2 µM) and increasing concentrations of nigratine or ferrostatin-1 (Fer-1) and incubated at 37 °C with 5% CO_2_ during 24 h in the IncuCyte S3 live-cell imaging and analysis system (Essen BioScience, Sartorius, Göttingen, Germany). Images were acquired using a 10X objective and 440–480 nm Excitation / 504–544 nm Emission filters, once hourly for up to 24 h.

### DPPH reduction assay

Antioxidant activity of compounds was determined by a DPPH (2,2-Diphenyl-1-picrylhydrazyl) reduction assay. DPPH was prepared at 15.85 µM in 90% methanol and added to several concentrations of compounds in 96-well plate (200µL per well). Tocopherol, a well-known antioxidant, was used as control. The reaction was conducted at room temperature for 30 min and absorbance was measured at 517 nm using the EnVision microplate reader (PerkinElmer, Waltham, MA, USA). The percentage of DPPH reduction was calculated by dividing the difference between the absorbance of DPPH and those of compounds by the absorbance of DPPH, and multiplying by 100.

### Statistical analyses

Data from a minimum of two experiments were expressed as means ± range; ± SD or ± SEM. Statistical analyses were done by ANOVA, Tukey's Multiple Comparison Test and Student’s *t*-test for two groups of data, and significance levels used are *P < 0.05, **P < 0.01, ***P < 0.001, ****P < 0.0001 by using GraphPad Prism6 software (GraphPad Software, San Diego, CA, USA).
